# Retraction: The Early Stage Formation of PI3K-AMPAR GluR2 Subunit Complex Facilitates the Long Term Neuroprotection Induced by Propofol Post-Conditioning in Rats

**DOI:** 10.1371/journal.pone.0196886

**Published:** 2018-05-01

**Authors:** The PLOS ONE Editors

The *PLOS ONE* Editors retract this article due to concerns about partial redundancy with previously published work and questions about the reported data that could not be resolved due to unavailability of primary supporting data.

Following publication, concerns were raised about similarities and partial redundancies between results in this *PLOS ONE* article [1] and an article published by the same group in the *Journal of Neurochemistry* [2]. Specifically, duplicate or replicate data for the Sham, I/R, and Propofol groups were reported in the following figures:

- Data are reported in duplicate in Figure 1 of the *PLOS ONE* article and in Figure 1 of the *J*. *Neurochem* article (panels A, B, C).
- Western blot data are duplicated in Figure 4 of the *PLOS ONE article* and Figure 4 of the *J*. *Neurochem* article.
- Data in Figure 2 of the *PLOS ONE* article replicate results presented in Figure 3 of the *J*. *Neurochem*. article.

In addition, questions were raised about the integrity and validity of some results reported in the *PLOS ONE* article:

- Regarding Figure 1, the authors explained that the *PLOS ONE* figure includes an extended data set relative to what was presented in *J*. *Neurochem*. (different n per group, different number of groups). However, the data appear largely the same for the three groups reported in both articles. Furthermore, it is unclear whether the expanded experimental and control groups include multiple cohorts of animals for which experiments were run at different times.
- In the Western blots of Figure 4, additional lanes are included in the *PLOS ONE* article that were not in the *J*. *Neurochem*. figure. The authors confirmed that they had removed the intralipid group lane and spliced in data for three additional groups (Wortmannin + sham, Wortmannin + I/R, Worthmannin + Propofol post-cond) when generating the *PLOS ONE* figure. It is unclear whether the spliced fragments were from the same experiments and blots as the other lanes in these figure panels.
- Some quantification data in the Figure 4 histograms are different though they were generated from the same source blots.

The authors cited the *J*. *Neurochem*. article in the Introduction and Methods sections of the *PLOS ONE* article, but they did not cite or acknowledge the reuse of published results in the *PLOS ONE* article. In response to questions about the overlap in data, the corresponding author explained that the two articles had different objectives and were conducted by different students but used some overlapping data. The authors also noted that the original data supporting the results in the *PLOS ONE* article are no longer available, and so we are unable to resolve outstanding questions about the aforementioned figures.

In light of these issues, the *PLOS ONE* Editors retract this article.

The journal editors have attempted to contact all co-authors on the manuscript by email. Haiyun Wang, Chenxu Wang, Zhiting Wen and Ai Zhu agreed with the retraction. Guolin Wang, Ying Wei, Chunyan Wang could not be reached.
